# Report of Case of Double Glioma Treated with X-Ray

**Published:** 1903-02

**Authors:** H. L. Hilgartner

**Affiliations:** Austin, Texas


For Texas Medical Journal.

### Report of Case of Double Glioma Treated With X-Ray.

BY H. L. HILGARTNER, M. D., AUSTIN, TEXAS.

The case in question is one of double glioma in a female child three and one-half years old. A competent oculist had correctly diagnosed and given advice for operation with the usual unfavorable prognosis. The parents, however, desiring another opinion brought the case to my office during the latter part of April. Examination confirmed the previous diagnosis, advice and prognosis; but in view of the desperate conditions I advised trial of X-ray treatment, though holding out only little encouragement to hope for success in saving the child's life.

The right eye had been first involved and the disease had progressed further in it than in the left eye; the growth had reached the pupil, filling the space occupied by the vitreous. The anterior chamber was nearly obliterated in consequence of the increased tension.

As usual, in cases so far developed, the great tension of the ball caused severe pain. In the left eye the growth could be easily discerned, and had already caused total blindness. The general appearance of the patient was cachectic.

The X-ray was applied by using lead plate perforated for both eyes to protect the face and head; the exposures were made once a day and lasted about fifteen minutes. After the second application the pain in right eye was relieved and the child seemed to rest comfortably at night. This treatment was continued without interruption eighty-four days, when the patient was allowed to be taken home for about two weeks, improvement justifying the rest. After returning to the sanitarium the patient was treated as before until December 20th for periods of two or three weeks with equal intermissions.

By December 20th careful examination revealed entire absence of any growth in the left eye, and the right had shrunken to two-thirds its normal size. The appearance of the latter was merely such as would be expected from the effects of the long maintained inflammation. The patient was in excellent health, having gained much in weight; color good, etc.

This case is to be kept under observation, but the results already obtained more than justify the present report, in view of the fact that it records the first experience (as far as I can ascertain) with X-ray treatment of glioma. The result is also interesting as perhaps tending to corroborate the theory that such cases of glioma originate in the retina and only later affect the optic nerve and brain.

